# Enhanced production of an anti-malarial compound artesunate by hairy root cultures and phytochemical analysis of *Artemisia pallens* Wall.

**DOI:** 10.1007/s13205-016-0496-5

**Published:** 2016-08-27

**Authors:** Zarna Pala, Vishnu Shukla, Anshu Alok, Subhash Kudale, Neetin Desai

**Affiliations:** 1 School of Biotechnology and Bioinformatics, D. Y. Patil University, Navi Mumbai, India; 2 Department of Biological Sciences, BITS Pilani, Pilani Campus, Pilani, Rajasthan India; 3 National Agri-Food Biotechnology Institute, Govt. of India, Mohali, Punjab India; 4 Amity Institute of Biotechnology, Amity University, Mumbai, India

**Keywords:** Artesunate, *Artemisia pallens*, Dhavanam, Malaria, Hairy roots, *Agrobacterium rhizogene*

## Abstract

Artemisinin and its derivatives are still one of the most effective drugs for the treatment of malaria. *Artemisia pallens* commonly known as Dhavanam, is an aromatic herb belonging to the family *Asteraceae*. Increasing the artemisinin content of *A. pallens* by genetic engineering would improve the availability of this much needed drug. In the present study, *Agrobacterium rhizogenes* (strain NCIM 5140) mediated genetic transformation of *Artemisia pallens* were carried out for hairy root induction. The effect of different media (Half MS, MS, MS along with BAP 0.5 mg/l and MS along with Kinetin 0.5 mg/l) and type of explants (leaf and stem) on hairy root induction and culture were also studied. Maximum transformation efficiency (70.0 %) was observed in case of stem explants when it was co-cultivated with *Agrobacterium rhizogenes* and kept on half strength MS media. Artesunate is a derivative of artemisinin, was quantified using HPLC from dried aerial extract and hairy roots. The content of artesunate in hairy roots was increased up to twofold as compared to aerial part of *Artemisia pallens*. The maximum amount of artesunate found in hairy roots was 5.62 ± 0.16 μg/g of dry weight. Apart from artesunate the other phytochemicals like alkaloids, polyphenols, and flavonoids are important because they impart the medicinal properties in this plant. Therefore, we have also quantified total alkaloids, flavonoids and polyphenolic content in the aerial part of the plants. The total alkaloids and flavonoids content were found 1.72 ± 0.00 mg/g dry weight in aqueous extract and 3.8 ± 0.00 mg/g in methanolic extract in terms of colchicine and rutin equivalents, respectively. Similarly, total phenolic content is 3.70 ± 0.01 mg/g in ethanolic extract in terms of tannic acid equivalent.

**Electronic supplementary material:**

The online version of this article (doi:10.1007/s13205-016-0496-5) contains supplementary material, which is available to authorized users.

## Introduction

Artemisinin is a highly potent anti-malarial drug found in the medicinal plant *Artemisia annua* (*A. annua*). World Health Organization recommends artemisinin and its derivatives for the treatment of malaria (White 2008). *Artemisia annua* is a plant species of genus *Artemisia* L. and is used as a traditional medicine for the treatment of malaria and other diseases in China. *A. pallens* also known as “Davana” is an important aromatic herb of genus *Artemisia* L. and is mostly found in southern region of India. These plants are industrially important due to its anti-microbial, insecticidal, antioxidant, and anti-malarial properties as well as perfumery compounds (Haider et al. 2014). *A. pallens* has been employed by the local people as a herbal medicine in curing many disease like diabetes and some skin infections (Haider et al. 2014). The leaves and flowers of the *A. pallens* yield an essential oil known as “oil of Davana”. Further, it also indicates the presence of artemisinin and its derivatives which has been used as a drug for the treatment of malaria (Shukla et al. 2015). Recently, *Agrobacterium tumefaciens* mediated genetic transformation of *A. pallens* has been optimized, which suggested that genetic engineering of *A. pallens* could be the effective strategy for the production of artemisinin and its derivatives (Alok et al. 2016). Artemisinin and its derivatives are produced only in the aerial parts (especially leaves and floral parts) of the plant whereas non significant amount or absent in roots of some species of Artemisia (Ferreira and Janick. 1996; Mannan et al. 2010; Wang et al. 2016).

Hairy root culture of plants using the *Agrobacterium rhizogenes*, the causative agent of hairy root disease in several plants, has emerged as an important technique for the production of secondary metabolites (Sivakumar et al. 2010; Sujatha et al. 2013). Transformed hairy root cultures are biochemically and genetically stable model for scale-up of pharmaceutically important natural products (Souret et al. 2003). Hairy roots have been reported to yield higher amounts of artemisinin than intact plant tissue and cell suspension cultures (Sivakumar et al. 2010; Dilshad et al. 2015a). Growth of hairy roots can be scaled up using bioreactors and hence they can be exploited for commercial production of secondary metabolites (Liu et al. 1998; Patra and Srivastava 2014). Various biotic and abiotic elicitors such as endophytic fungi (Wang et al. 2001a), red light (Wang et al. 2001b) and methyl jasmonate (Baldi and Dixit 2008) have been reported to regulate cell metabolism of hairy root for enhanced production of artemisinin.

Among the plethora of phytochemicals, terpenes (terpenoids or isoprenoids) constitute the largest and most diverse class of specialized metabolites. Terpenoids, are the plant secondary metabolites which play an important role in plant–microbe, and plant–plant interactions (Dudareva et al. 2006). Terpenes are one of the largest groups of natural products; more than 25,000 terpene structures have been reported in different plant species (Gershenzon and Dudareva 2007). Terpene compounds have many functional roles in plants such as in photosynthesis (plastoquinones, chlorophylls, carotenoids), respiration (ubiquinone) as well as in growth and development of plant (sterols, cytokinins, gibberellins, abscisic acid, brassinosteroids) (Pulido et al. 2012). Natural bioactive compounds like alkaloids, flavonoids and polyphenols are the most important secondary metabolites in plants having properties that affect appearance, taste, odor and oxidative stability (Singh 2012). These compounds posses various important biological properties. Therapeutic potential of the extract of Artemisia species is directly related to the total polyphenolic and flavonoid content in those plants.

Artemisinin is the drug of choice for the treatment of malaria and other diseases (White 2008). The highest artimisinin content has been reported in the leaves of *A. annua* (0.44–1.00 %), a Chinese variety of Artemisia plants (http://www.mmv.org/) (Mannan et al. 2010). It was introduced and cultivated in different regions of India, but artemisinin production was decreased (Singh et al. 1988; Baldi and Dixit 2008). There is a great concern that the artemisinin production at the current rate will not meet the increasing demand by the pharmaceutical industry. In past, various efforts have been made to enhance the level of these molecules in plants by the genetic engineering approach (Farhi et al. 2011). There are various strategies which are now being used to meet the increasing demand of artemisinin (Durante et al. 2011; Farhi et al. 2011; Singh et al. 2016).

Therefore, present study was aimed to investigate efficacy of the hairy root induction in cultures of *A. pallens*, an Indian Artemisia species. We quantified artesunate, an important derivative of artemisinin in the hairy roots of *A. pallens* and aerial extract of plant. To the best of our knowledge, this is the first report of *A. rhizogenes* mediated genetic transformation of *A. pallens*. Further, we also quantified the total content of alkaloids, flavonoids and phenolic comounds in different extracts prepared in aqueous, ethanolic and methanolic solvents.

## Materials and methods

### Plant material, sterilization and media preparation

Seeds of *A. pallens* were collected from Kolhapur, Maharashtra. Seeds were rinsed twice with distilled water; and then sterilized with 0.1 % mercuric chloride for 5 minutes. Finally, the seeds were rinsed with sterile distilled water for 3–4 times. The seeds were germinated on MS medium with 3 % sucrose and 0.8 % agar and pH maintained at 5.8.

### Bacterial strain and culture conditions

*A. rhizogenes* strain NCIM 5140 (ATCC 15834) was obtained from the National Chemical Laboratory, Pune, India and was used for the induction of hairy root. The bacterial culture was revived and maintained on YEB agar medium. Loop full of bacterial colonies were inoculated in 100 ml of liquid YEB medium and the culture was kept on a rotary shaker (100 rpm) at 30 °C overnight till the O.D. at 600 nm was about 0.8.

### *A. rhizogenes* mediated genetic transformation

Stem and leaves were dissected from in vitro germinated seeds and used as explants. The explants were pre-cultured for 3 days on MS basal medium (Murashige and Skoog 1962). Overnight grown culture of *A. rhizogenes* was centrifuged at 5000 rpm for 10 minutes at 26 °C. Pellet was re-suspended in 10 ml liquid MS medium and the O.D. at 600 nm was adjusted about 0.6. The pre-cultured explants were now co-cultured with *A. rhizogenes* grown in the flask. The flask was wrapped with foil and kept on shaker at 90 rpm for 30 minutes. Explants were blotted dry on a sterile filter paper and transferred on MS basal media and kept in dark. After 3 days of co-cultivation, the explants were transferred to medium containing 400 mg/l cefotaxime to kill the residual *Agrobacterium*. The explants were again sub-cultured on medium containing 300 mg/l cefotaxime after a week. The concentration of cefotaxime was reduced gradually to 100 mg/l.

### Optimization of hairy root induction and growth on different media

The co-cultivated explants were incubated on different media [Half MS, MS, MS supplemented with 6-Benzylaminopurine (BAP) 0.5 mg/l and MS supplemented with Kinetin 0.5 mg/l] for proper induction and growth of hairy roots. The MS basal medium contained 2 mg/l thiamine hydrochloride, 5 mg/l nicotinic acid, 10 mg/l pantothenic acid, 30 g/l sucrose, and 100 mg/l *myo*-inositol. All media were solidified with 0.8 % agar and the pH was adjusted to 5.8 with 1 N NaOH or 1 N HCl prior to sterilization at 121 °C for 20 minutes. All the cultures were maintained in complete darkness at 26 ± 1 °C. The response of explants (leaf and stem) in different media was recorded. The final and bulk hairy root induction was performed on the best responded media. The 100 mg/l cefotaxime was also used in all different media combinations to eliminate the residual bacteria.

### PCR analysis of hairy roots

The Polymerase chain reaction (PCR) was used to detect the Ri T-DNA integration into genome of *A. pallens* (Eppendorf Mastercycler^®^, Germany). Genomic DNA from putative transgenic hairy roots as well as non-transgenic roots (in vitro plants roots) was extracted as per manufacturer protocol using HiPurA^™^ Plant Genomic DNA Miniprep Purification Kit (Himedia, India). The transgenic nature of the roots was confirmed by amplification of *mannopine synthase* 1′ (*mas* 1′) sequence using *mas*(*For*): 5′CGGTCTAAATGAAACCGGCAAACG3′ and *mas*(*Rev*) 5′GGCAGATGTCTATCGCTCGCACTCC3′ as mentioned by (Telke et al. 2011). Further confirmed by amplification of *rolC* gene using *rolC*(*For*):5′ATGGCTGAAGACGACCTGTGTT3′ and *rolC*(*Rev*):5′TTAGCCGATTGCAAACTTGCTC3′ as mentioned by (Jha et al. 2013). The PCR reactions were carried out in a total 25 µl volume and consisted of 50 ng of DNA, 2.5 μl 10× taq DNA polymerase buffer, 50 μM dNTPs, 0.2 μM primers and 0.5 U Taq DNA polymerase (Banglore Genei Pvt. Limited). DNA amplification was performed on an Eppendorf thermal cycler with following PCR cycle conditions: 94 °C for 2 minutes, 94 °C for 30 s, and 55 °C for 30 s, 72 °C for 90 s and final extension at 72 °C for 5 minutes. Amplification products were separated by electrophoresis on 1 % agarose gel in 1× TBE buffer, stained with ethidium bromide and visualized under UV trans-illuminator.

### Extraction and estimation of artesunate using HPLC

The extraction process for the HPLC analysis of samples was optimized with different solvents. We choose methanol: water combination for the extraction of our samples as it gave the best response with both standard and the sample. The powdered plant material was repeatedly extracted three times with methanolic extract (methanol: water combination) on an orbital shaker for overnight incubation at room temperature. The pooled plant extract was centrifuged at 10,000 rpm for 10 min at room temperature and the supernatant was collected and filtered before HPLC analysis. The HPLC system equipped with a Waters 1525 binary pump and Waters 2487 dual wavelength absorbance detector was used for artesunate quantification. A known amount of artesunate (Sigma Aldrich) was dissolved in mobile phase, centrifuged and supernatant was injected in HPLC. The HPLC analysis of the samples was carried out as previously described protocol (Affum et al. 2013). The reverse phase C18 column of dimensions 0.39 mm × 150 mm (i.d.) and 5 μm particle size was used in the analysis. Before analysis, the column was equilibrated with a mobile phase prepared from acetonitrile and phosphate buffer at pH 3 (44:56 % v/v). 20 μl of the sample was injected into HPLC instrument with a flow rate of 1.0 ml/min for 10 min using an isocratic elution. The chromatograms of each sample were recorded at 216 nm. Artesunate content in the extracts was determined by comparing the peak areas of the sample with those of standard artesunate.

### Extraction of alkaloids, flavonoids and poyphenols

Aerial part of *A. pallens* weighed 10 g and then it was crushed to fine powder in mortar and pestle using liquid nitrogen. Three different extracts were prepared in 100 ml 99.9 % ethanol, 99.9 % methanol, and disstiled water. This mixture of 10 g powdered plant material and 100 ml of its respective solvent was kept overnight for incubation. The mixture was then centrifuged at 10,000 rpm for 10 min at room temperature. The supernatant was collected in different tubes and labelled correctly.

### Determination of total alkaloids

Alkaloid content of the different extracts was estimated using colchicine as standard and protocol followed as per mentioned by (Singh et al. 2004). Ethanol was dissolved and prepared 0.05 M 1,10-Phenanthroline monohydrate and 0.5 M HCl was used to prepare 0.025 M FeCl_3_. From each solution 1 ml were added to 1 % extract and final volume was made up to 10 ml with distilled water. The tubes were incubated at 70 ± 2 °C for 30 min. O.D. was taken at 510 nm using UV–VIS spectrophotometer. Once the O.D. is obtained then the standard graph for alkaloids is prepared and the concentrations of the samples are calculated following the Beer Lambert’s Law.

The concentration of alkaloids in the test extracts was calculated from the calibration plot and expressed as milligrams of colchicines equivalent per gram dry weight of sample (mg CE/g).

### Measurement of total flavonoids

Rutin was used as the standard compound for the estimation of flavonoids and protocol used by (Zhishen et al. 1999) was followed. A known volume of 1 % extract was placed in a 10 ml volumetric flask containing 3 ml distilled water and 0.3 ml NaNO_2_ (1:20) were added. After 5 min later, 3 ml AlCl_3_ (1:10) were added. Finally after 6 min, 2 ml 1 M NaOH was added and the total volume was made up to 10 ml with distilled water. The solution was mixed well again absorbance was measured against a blank at 510 nm using UV–VIS spectrophotometer. Rutin was used to make the standard calibration curve by making different dilutions. The total flavonoid content of each sample was determined from the standard curve of Rutin. The total flavonoid content was expressed as milligrams of rutin equivalent per gram dry weight of sample (mg RE/g).

### Determination of total poyphenols

The total phenolic content of extract *A. pallens* was determined by using Folin-Ciocalteu reagent with slightly modified method as prescribed by (Ainsworth and Gillespie 2007). Different dilution of tannic acid was used for standard calibration curve. A volume of 0.5 ml of the 1 % extract was mixed with 2 ml of the Folin-Ciocalteu reagent (diluted 1:10 with de-ionized water) and finally neutralized with 4 ml of sodium carbonate solution (7.5 % w/v). The reaction mixture was incubated for 30 min at room temperature. The absorbance of the resulting blue color was measured at 765 nm using double beam UV–VIS spectrophotometer. The total polyphenolic contents were determined from the linear equation of a standard curve prepared with tannic acid. The total flavonoid content was expressed as milligrams of tannic acid equivalent per gram dry weight of sample (mg TAE/g).

### Quantification of different polyphenols using HPLC

Separation of tannic acid, cathacol, ferulic acid and vaniline in the crude extract of *A. pallens* was achieved on HPLC system equipped with a PDA Detector. Xterra MSC-18 column (7.8 × 100 mm, 5 µm) with octadecylsilane as a solid support and 600e Multi Solvent Delivery System from Waters (USA) was used to separate the components. The mobile phase consists of methanol + acetonitrile:phosphate (30:15:55, v/v) (pH 3.5) and flow rate of the mobile phase was kept at 1.0 ml/min with isocratic elution. A linear gradient elution was used and detection was done at 280 nm in this method. The separated alkaloids, flavonoids and phenolic content were initially identified by a direct comparison of their retention times with those of standards. The contents of all compounds were calculated from the peak areas of HPLC chromatograms from the three replicate samples and the output was given in the units of ppm. The results were converted from ppm to mg/g. The data were analysed and processed using the installed Empower 2 software.

## Results and discussion

### Induction and establishment of hairy root cultures

**Fig. 1:**
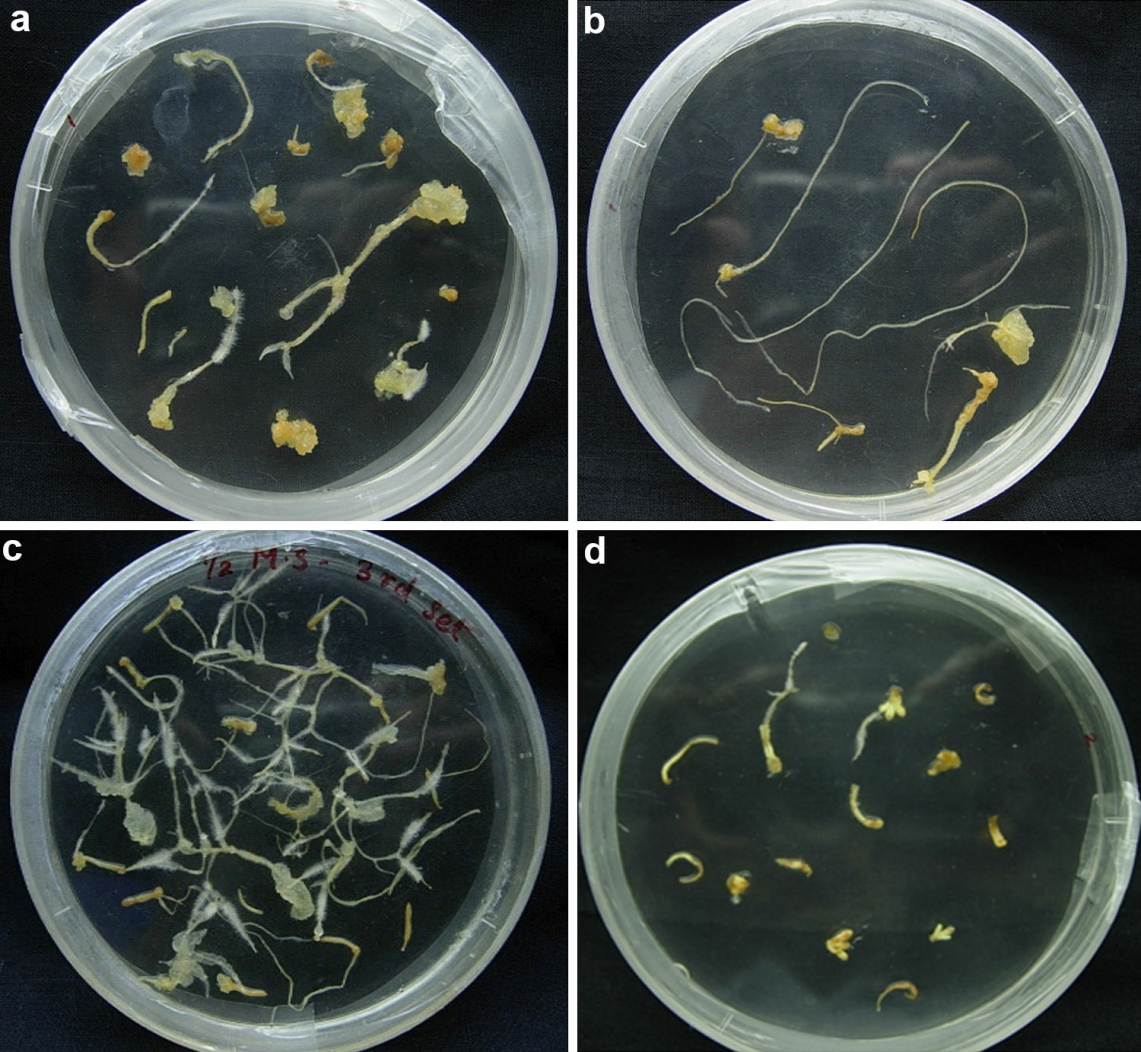
Hairy root response after 30 days of co-cultivation on different media composition. **a** Leaf as explants on MS media, **b** stem as explants on MS media, **c** stem as explants on half strength MS media and **d** leaf as explants on MS media supplemented with 0.5 mg/l Kinetin

*Agrobacterium rhizogenes* strain, NCIM 5140 was used to determine the transformation efficiency towards hairy root induction in *A. pallens*. Leaf and stem of 4 weeks old in vitro germinated seedling were used as explants for hairy root induction. After co-cultivation of explants with *A. Rhizogenes,* the explants were kept on different media. The transformation efficiency of hairy root induction on different media is shown in Table 1. The best response of hairy root induction was found in all media when stem was co-cultivated as compared to leaf. The some leaf explants turned to callus like structure on MS media with thick and short hairy roots (Fig. 1a) whereas roots raised from stem were long and thin (Fig. 1b). Maximum transformation efficiency (70.0 %) was observed in case of stem explants when it was kept on half strength MS media for hairy root induction (Fig. 1c). The response of hairy root induction was very less (30.75 %) in case of leaf when it was incubated on MS media supplemented with 0.5 mg/l Kinetin (Fig. 1d). In all combinations hairy roots obtained from stem explants were very thin, long and appeared very weak whereas, roots obtained from the leaf explants were thick and short. After 5 weeks of growth, roots obtained from both the explants appeared same. Influence of *A. rhizogenes* on hairy root induction frequency has been documented earlier in several plant species. In *A. vulgaris* maximum of 92.6 % transformation frequency was observed in leaf explants followed by 64.3 % in node and 38.1 % in shoot tip using strain, A_4_GUS (Sujatha et al. 2013). Transformation efficiency depends upon type of explants and strain of *A. rhizogenes*. However, NCIM 5140 strain of *A. rhizogenes* was widely used for hairy root induction in many plants such as Linum album (Baldi et al. 2008), *Abrus precatorius* L. (Karwasara and Dixit 2009), *Helianthus annuus* L. (Jha et al. 2013) and *Sesuvium portulacastrum* L. (Lokhande et al. 2015).

**Table 1:** Frequency of hairy root induction on different explants and media of *A. pallens* by *Agrobacterium rhizogenes* strains, NCIM 5140

Type of explants and different media	No. of explants infected	No. of explants transformed	Transfomation efficiency (%)
Leaf (half MS)	30	15	50.00
Stem (half MS)	50	35	70.00
Leaf (MS)	46.5	25	53.76
Stem (MS)	27	17	59.25
Leaf (MS + 0.5 mg/l BAP)	90	30	33.33
Stem (MS + 0.5 mg/l BAP)	80	50	62.5
Leaf (MS + 0.5 mg/l Kinetin)	65	20	30.75
Stem (MS + 0.5 mg/l Kinetin)	60	40	66.66

### PCR based analysis of transgenic hairy roots

**Fig. 2:**
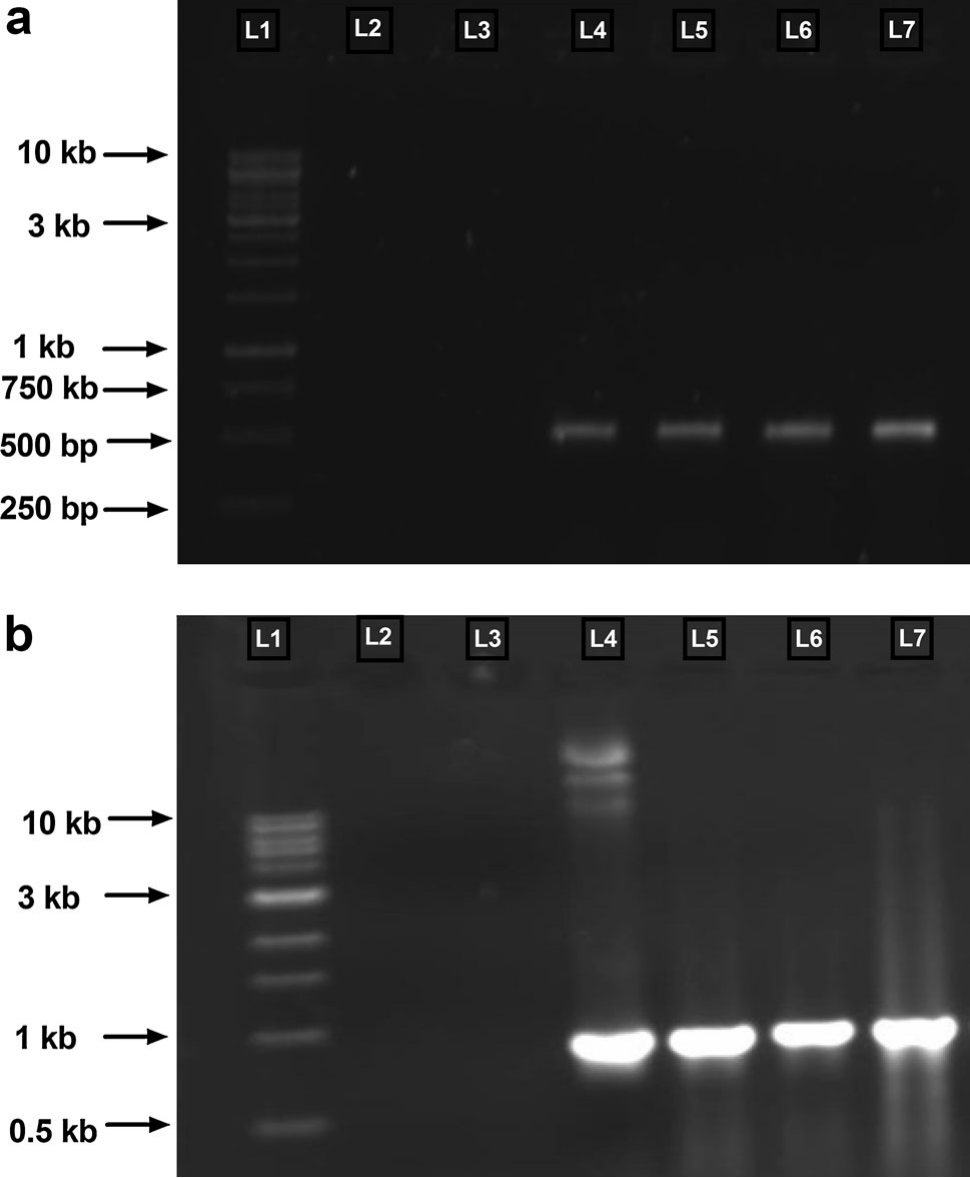
PCR detection of *rol* C and *mas*1′gene in hairy roots. **a** *Lane L1*, GeneRuler 1 kb DNA Ladder;* lane L2*, water control;* lane L3*, non-transformed roots (negative control);* lane L4*, *rol* C positive control (Ri plasmid);* lane L5* to *L7*, *rol* C from transformed hairy roots. **b** *Lane L1*, NEB 1 kb DNA Ladder;* lane L2*, water control;* lane L3*, non-transformed roots (negative control); *lane L4*, *mas*1′positive control (Ri plasmid); *lane L5* to *L7*, *mas*1′from transformed hairy roots

The transgenic nature of hairy roots was confirmed by PCR amplification of DNA from *A. pallens* hairy roots with *mas* gene specific primers, which showed the expected fragment size of 970 bp (Fig. 2b). 970-bp fragment amplification was observed in all the transformants; while it was absent in the non-transformed control roots. *Rol C* gene specific primers were also used for further confirmation of integration of T-DNA from the *A. rhizogenes* into the hairy roots. PCR analysis showed amplification of 500 bp band of *Rol C* gene integrated in hairy roots obtained from leaf and stem explants (Fig. 2a). Genomic DNA of strain NCIM 5140 was used as positive control where as negative control (non-transformed roots) there was no amplification (Fig. 2a, b). In Ri plasmids, the T-DNA region consists of two parts: T_L_-DNA and T_R_-DNA separated by a non-transferable DNA sequence. T_R_ DNA consists of mas 1′ where as T_L_-DNA consist *rol* gene, which is is essential to induce hairy roots (Hansen et al. 1991). Therefore we used both gene specific primers to confirm transgenic nature of our hairy root culture. Similarly, both this primes are used to demonstrate transgenic nature of *Sesuvium portulacastrum* L. by the same strain *A. Rhizogenes* (Lokhande et al. 2015).

### Determination of artesunate in hairy roots through HPLC coupled with PDA

The HPLC chromatograph of standard and artesunate from the dried aerial part and hairy root of *A. pallens* is depicted in Supplementary Fig. 1. The results confirmed a considerable amount of artesunate (5.62 ± 0.16 μg/g of DW) in the hairy roots where as in dried aerial part and (1.88 ± 0.10 μg/g of DW) was found Table 3. The content of artesunate in *A. annua* it was approx 10 μg/g (Dilshad et al. 2015a) whereas in *A. carvifolia* is found 2.24 μg/g (Dilshad et al. 2015b). So, it can be concluded that the hairy roots are a good source of accumulation of secondary metabolites, in this case artesunate which is an important anti-malarial compound. The accumulation of high content of artesunate in hairy roots of *A. pallens* could be due to the effect of *rol* gene integration on the secondary metabolite production as reported in many plant species (Bulgakov 2008). The transformation of *rol ABC* gene construct in *A. dubia* enhanced over production of artemisinin in i.e., up to tenfold than individual *rol* genes as we observed six- to sevenfold increase (Kiani et al. 2012). Recently, the transformation of *A. annua* with *rol B* gene showed two- to ninefold increase in artemisinin in different transgenic lines. They have also quantified artesunate in different transgenic line and they found 4- to 12-fold increase in artesunate content (Dilshad et al. 2015a). The artemisinin and its derivative such as artesunate, and dihydroartemisinin etc. are very effective for malaria, and therefore recently, theses metabolites were isolated and quantified from *A. carvifolia* Buch. These metabolites were quantified in the shoots extract of *A. carvifolia* at the following concentrations: artemisinin (8 μg/g), artesunate (2.24 μg/g), dihydroartemisinin (13.6 μg/g) and artemether (12.8 μg/g). Genetic transformation of *A. carvifolia* using *rol B* and *C* genes increased three- to sevenfold artemisinin, three- to tenfold for artesunate and 2.6- to 4-fold for dihydroartemisinin and artemether in transgenics lines (Dilshad et al. 2015b).

### Quantification of alkaloids, flavonoids and phenolics content

Standard curve prepared was used for the determination of total alkaloids, flavonoids and phenolic content of *A. pallens* using different concentrations of colchicine, rutin and tannic acid respectively. The total alkaloids, flavonoids and phenolic content in different solvent of *A. pallens* have been presented in Table 2. Observation shows that the total alkaloids content was almost similar, however highest alkaloids content was found in aqueous extract (1.72 ± 0.00 mg/g CE of dried extract). The concentration of flavonoids is significantly high (3.8 ± 0.00 mg/g RE of dried extract) in methanolic extract as compared to ethanolic extracts (2.13 ± 0.56 mg/g RE of dried extract). Similarly, total phenolic content is highest in the ethanolic extract (3.7 ± 0.01 mg/g TAE of dried extract) followed by methanolic and significantly lower in aqueous extract (3.0 ± 0.00 mg/g TAE of dried extract).

**Table 2:** The total alkaloids, flavonoids and phenolics content present in different extracts of *A. Pallens*

Parameters	Aqueous extract	Ethanolic extract	Methanolic extract
Total alkaloids content (mg/g CE)	1.72 ± 0.00	1.68 ± 0.01	1.67 ± 0.00
Total flavoinds content (mg/g RE)	3.70 ± 0.1	2.13 ± 0.56	3.80 ± 0.00
Total phenolics content (mg/g TAE)	3.00 ± 0.00	3.70 ± 0.01	3.20 ± 0.01

Values are expressed as mean ± SE of three replicates

Further, the different phenolics compounds such as tannic acid, catechol, vanillin, and ferulic acid was also quantified in plant extract using HPLC and the chromatograph is depicted in Supplementary Fig. 2. Phenolics are aromatic benzene ring compounds that have one or more hydroxyl groups. These individual phenolic compounds were extracted in methanolic extract (Table 3). The content of Tannic acid (3. 41 ± 0.04), Cathacol (5.37 ± 0.03) and Ferulic Acid (1.94 ± 0.00); whereas Vaniline was not detected (Table 4).

**Table 3:** Quantification of artesunate (μg/g of DW) using HPLC of dried areial part and hairy roots of *A. pallens*

Tissue type	Content of artesunate (μg/g of DW)
Hairy roots	5.62 ± 0.16
Aerial parts	1.88 ± 0.10

**Table 4:** Quantification of different polyphenols using HPLC of dried areal part of *A. pallens*

Parameters	Methanolic extract
Tannic acid (μg/g)	3.41 ± 0.04
Cathacol (μg/g)	5.37 ± 0.03
Ferulic acid (μg/g)	1.94 ± 0.00
Vaniline (μg/g)	ND

## Conclusion

The overall goal of this study was to induce hairy roots in *A. pallens* and quantified the content of secondary metabolites produced in hairy roots and areal parts. *A. pallens* are of great medicinal importance in *diabeteus mellitus*. Apart from these the presence of artesunate also confirmed it can use for as an alternative system for the production of artemisinin. Further the total content of alkaloids flavanoids, and phenolic compounds was also quantified. These natural bioactive compounds like alkaloids, flavonoids and phenols are the important secondary metabolites in plants. These compounds have intrinsic properties that affect appearance, taste, odor and oxidative stability of plant based foods. To the best of our knowledge this is the first report on hairy root induction and quantification of artesunate from *A. pallens.* This study provides basic platform to initiate large scale production of artemisinin and its derivatives and needs further standardization for industrial application.

## Electronic supplementary material

Below is the link to the electronic supplementary material.

Supplementary material 1 (JPEG 1282 kb)

Supplementary material 2 (JPEG 1236 kb)
