# Supplementary figures and images for: Enhanced production of an anti-malarial compound artesunate by hairy root cultures and phytochemical analysis of Artemisia pallens Wall
Source: 3 Biotech. 2016 Aug 27;6(2):182. doi: 10.1007/s13205-016-0496-5 (PMC5002272; doi:10.1007/s13205-016-0496-5)

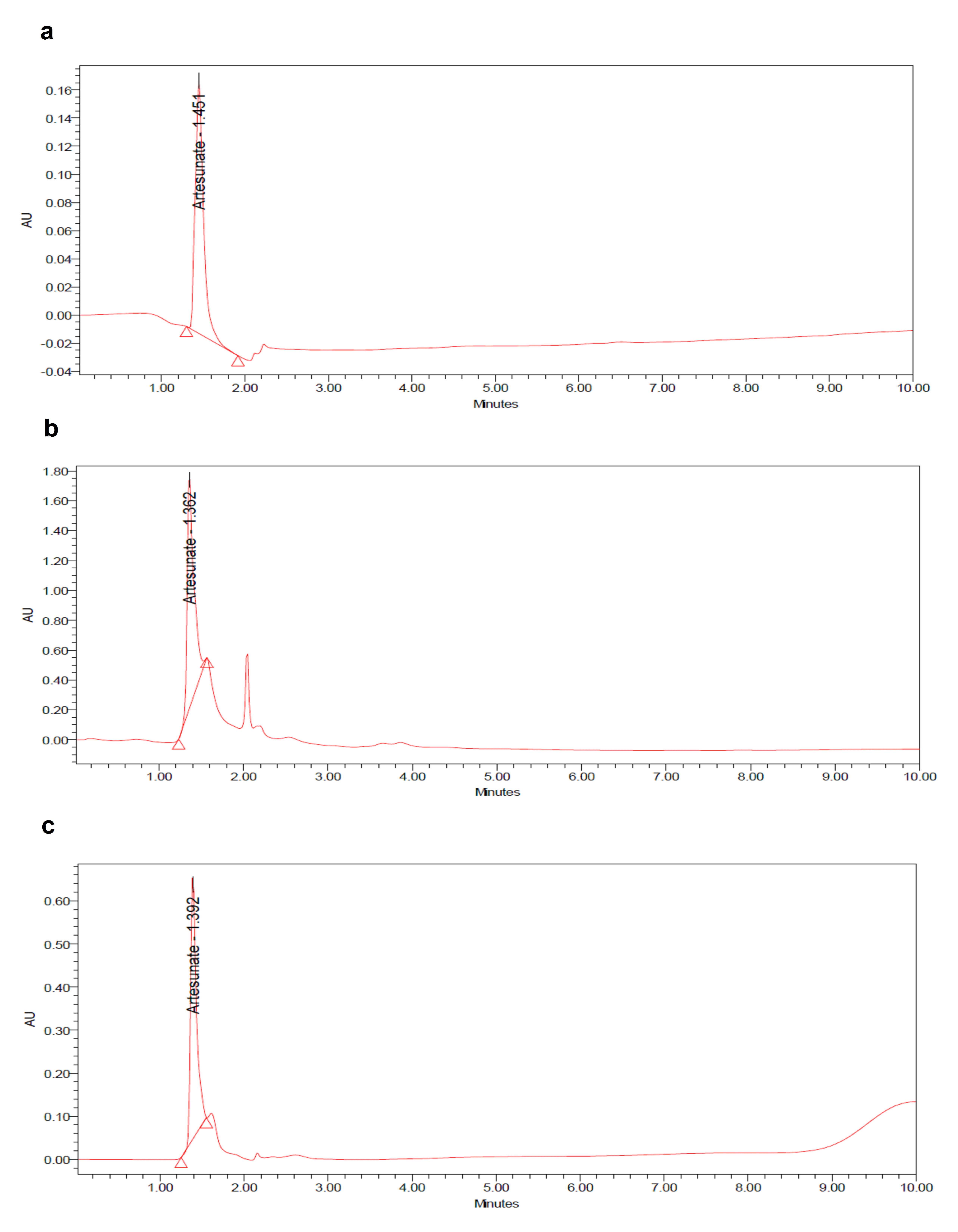

Supplement: Supplementary file 1 — Supplementary material 1 (JPEG 1282 kb) [file 13205_2016_496_MOESM1_ESM.jpg]

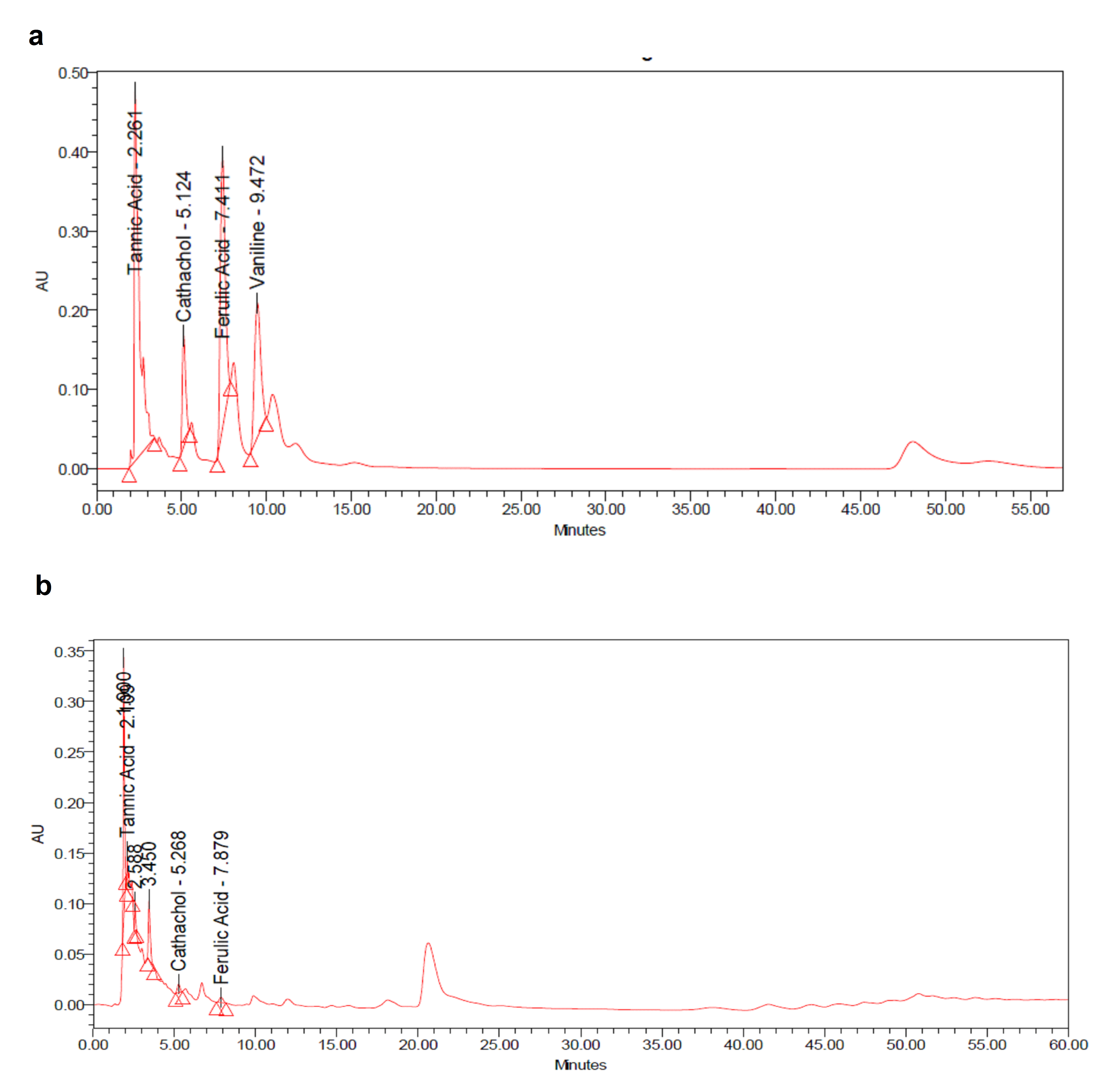

Supplement: Supplementary file 2 — Supplementary material 2 (JPEG 1236 kb) [file 13205_2016_496_MOESM2_ESM.jpg]
